# A New Chemical Approach to Human ABO Histo-Blood Group Type 2 Antigens

**DOI:** 10.3390/molecules19010414

**Published:** 2013-12-31

**Authors:** Atsushi Hara, Akihiro Imamura, Hiromune Ando, Hideharu Ishida, Makoto Kiso

**Affiliations:** 1 Department of Applied Bioorganic Chemistry, Faculty of Applied Biological Sciences, Gifu University, 1-1 Yanagido, Gifu-shi, Gifu 501-1193, Japan; E-Mails: r8101036@edu.gifu-u.ac.jp (A.H.); hando@gifu-u.ac.jp (H.A.); ishida@gifu-u.ac.jp (H.I.); 2 Institute for Integrated Cell-Material Sciences (WPI-iCeMS), Kyoto University, Yoshida Ushinomiya-cho, Sakyo-ku, Kyoto 606-8501, Japan

**Keywords:** blood group antigen, oligosaccharide, glycosylation, Heyns rearrangement

## Abstract

A new chemical approach to synthesizing human ABO histo-blood type 2 antigenic determinants was developed. *N*-Phthaloyl-protected lactosaminyl thioglycoside derived from lactulose via the Heyns rearrangement was employed to obtain a type 2 core disaccharide. Use of this scheme lowered the overall number of reaction steps. Stereoselective construction of the α-galactosaminide/galactoside found in A- and B-antigens, respectively, was achieved by using a unique di-*tert*-butylsilylene-directed α-glycosylation method. The proposed synthetic scheme provides an alternative to existing procedures for preparing ABO blood group antigens.

## 1. Introduction

**Figure 1:**
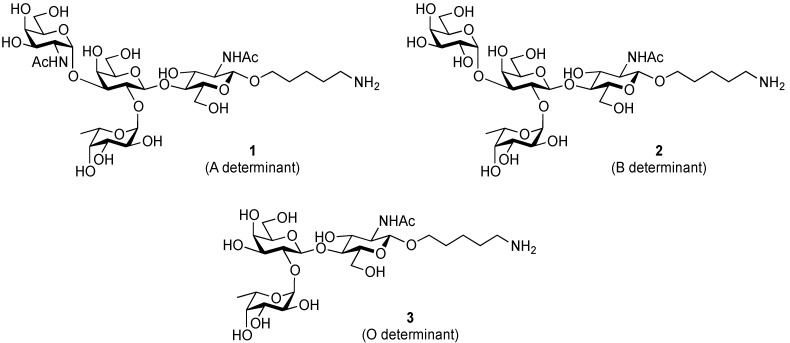
Structure of the target ABO blood-group type 2 antigens.

ABO histo-blood group antigens are expressed on red blood cells and are widely distributed in various tissues such as the vascular endothelium, where they are displayed on plasmalemmal glycoproteins and glycolipids by attachment to sugar residues that terminate *N*-linked, *O*-linked, and lipid-linked glycans [1,2]. The A, B, and O group antigens are defined by the GalNAcα(1-3)[Fucα(1-2)]Gal, Galα(1-3)[Fucα(1-2)]Gal, and Fucα(1-2)Gal glycan structures, respectively. These antigens can be further divided into six subtypes based on linkage arrangement: type 1, ABO-β(1-3)GlcNAcβ; type 2, ABO-β(1-4)GlcNAcβ; type 3, ABO-β(1-4)GalNAcα; type 4, ABO-β(1-3)GalNAcβ; type 5, ABO-β(1-3)Galβ; and type 6: ABO-β(1-4)Glcβ [3,4]. Since the discovery of ABO antigens over a century ago [5], many biological phenomena associated with them have been found, for example: immune response in blood transfusion and organ transplantation [6,7]; susceptibility to certain diseases in individuals with a particular ABO phenotype [8,9,10]; function as a receptor for pathogens such as *Campylobacter jejuni* [11], *Helicobacter pylori* [12], and Norwalk virus [13,14]; and aberrant expression in the oncogenesis of various organs [15,16]. However, little progress has been made in elucidating their physiological behavior at the molecular level because of a lack of pure materials for scientific research. We envisioned that chemically synthesized pure samples would enable a range of studies on the physiological and pathological implications of ABO group antigens. The chemical synthesis of type 1 glycans has been reported by several groups [4,17,18,19,20,21,22,23], but reports on the synthesis of type 2 glycans have been limited [4,24,25,26]. Also, it should be noted that synthetic studies of ABO antigens were first reported by Lemieux's group and they then focused on Lewis antigens [27,28,29,30,31]. The goal of our research is therefore to develop a facile synthetic route to ABO histo-blood group antigens, particularly type 2 glycans. Here we describe a new chemical approach to ABO group type 2 antigens with a pentylamine linker (**1‒3**; Figure 1), which are expected to be useful in future biological studies.

## 2. Results and Discussion

**Scheme 1:**
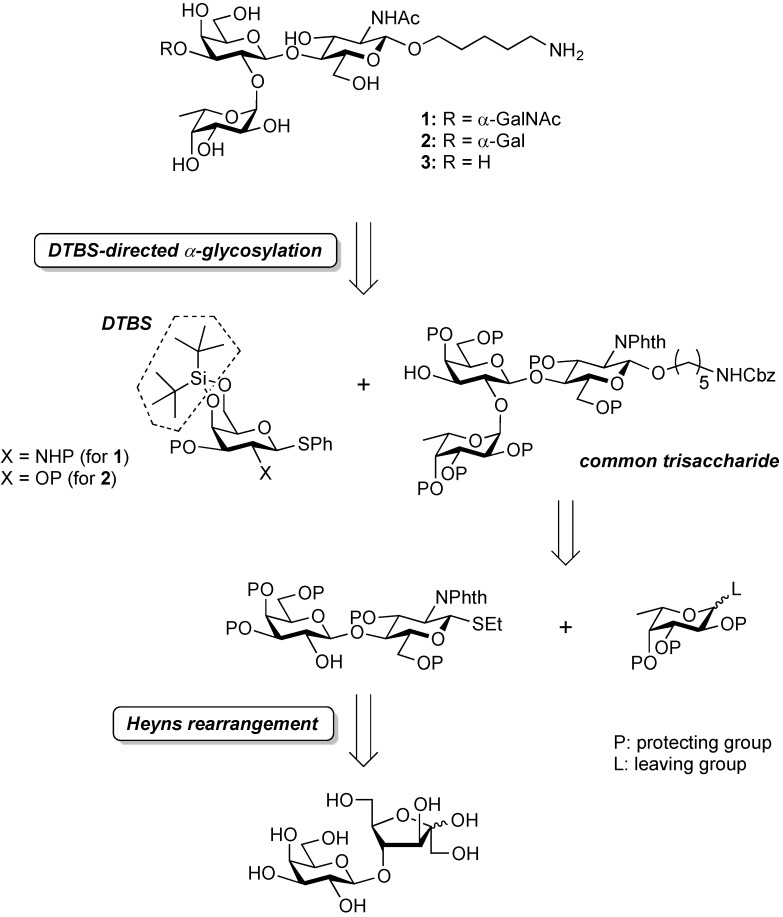
Retrosynthetic analysis of target compounds.

The typical procedure for synthesizing ABO blood group antigens is stepwise assembly of the monosaccharide unit, which requires a laborious protection/deprotection strategy for the multistep preparation of both monosaccharide donor and acceptor. To improve accessibility to those antigens, we designed a unique synthetic route to the target ABO group type 2 antigenic oligosaccharides. As shown in Scheme 1, our synthetic strategy involves two key reactions: (1) the Heyns rearrangement for simple preparation of *N*-acetyl-lactosamine (4-*O*-β-d-galactopyranosyl-d-*N*-acetyl-glucosamine), a type 2 core disaccharide; and (2) di-*tert*-butylsilylene (DTBS)-directed α-galactosaminylation and α-galactosylation for the formation of A and B determinants, respectively.

**Scheme 2:**
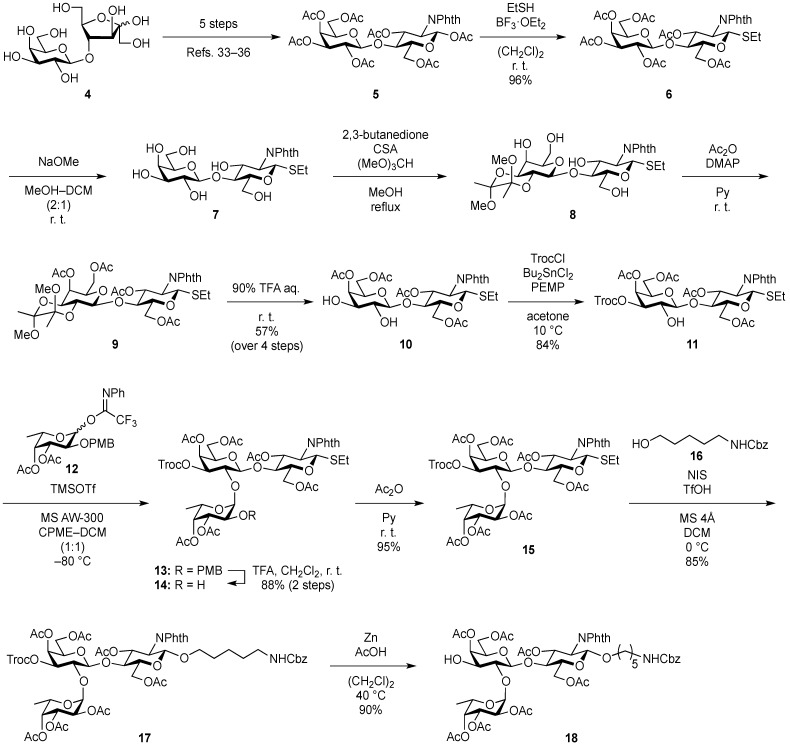
Synthesis of the common trisaccharide unit.

The Heyns rearrangement is known to be effective for obtaining a lactosamine derivative by simple manipulation starting from lactulose (4-*O*-β-d-galactopyranosyl-d-fructose). This reaction was originally developed for converting ketoses into the corresponding 2-amino-2-deoxyaldoses [32]. We hoped that the use of lactulose (**4**) as an alternate starting material would allow us to minimize the number of reaction steps as well as to reduce the time and effort needed. Additionally, lactulose is a relatively inexpensive and commercially available sugar. Recently, Wrodnigg and co-workers reported an improved Heyns rearrangement procedure, which was much more practical than the original procedure [33,34]. Other groups have recently reported even more practical protocols suitable for large-scale synthesis [35,36]. In the present study, we followed these procedures to obtain lactosamine derivative **5** [37] as a key building block. Compound **5** was efficiently prepared in five steps (Scheme 2) by methods in the literature [33,34,35,36]. Conversion of peracetate derivative **5** into thioglycoside form was performed in the presence of ethanethiol and BF_3_·OEt_2_ in 1,2-dichloroethane to give ethylthioglycoside **6** in 96% yield. The ethylsulfinyl group was selected in consideration of its solubility in MeOH, which was used in the next step. A phenylsulfinyl group in place of the ethylsulfinyl group resulted in poor solubility in MeOH, leading to a poor results in the deacetylation reaction. After removal of all acetyl groups in **6**, hydroxyl groups at the C2 and C3 positions of the galactose residue were simultaneously protected as a butanediacetal (BDA) [38] to afford compound **8**. In this reaction, a regioisomer of **8**, namely, a 3,4-*O*-BDA-protected by-product, was formed and these regioisomers were separated by silica gel column chromatography. However, small amounts of impurities could not be separated from **8**. Acetylation of **8** along with contaminants and subsequent hydrolysis of the BDA group afforded diol **10** as the sole product in 57% yield over the four operations. The tin-mediated selective acylation developed by Muramatsu [39] was then applied to selectively protect the C3′-OH group by the Troc group, giving the disaccharide acceptor **11** in 84% yield. Another procedure for selective protection of the C3′-OH group by treatment of TrocCl with pyridine in CH_2_Cl_2_ at lower temperature (−40 °C) gave **11** in somewhat lower yield (76%). For next glycosylation, the fucosyl *N*-phenyltrifluoroacetimidate **12** was designed to increase both reactivity and stability as a fucose donor. The previously used fucosyl donor, 2,3,4-tri-*O*-benzyl-protected fucosyl imidate, could be served as a good fucosyl donor, but was relatively unstable under glycosylation conditions due to its armed feature. Chemo-selectively removable PMB group was chosen as a protecting group at C2 position and electron-withdrawing acetyl groups at C3 and C4 were incorporated to suppress the armed feature by the PMB group, which could lead to stabilization of the donor. Furthermore, a more stable *N*-phenyltrifluoroacetimidate group compared to a trichloroacetimidate group was used as a leaving group [40,41]. The glycosylation of **11** with **12**, which was derived from a known fucose derivative [42] and was promoted by TMSOTf in a mixed solvent system of cyclopentylmethyl ether (CPME)–dichloromethane (1:1) [43] at −80 °C, provided trisaccharide **13**. Small amounts of contaminates remained after column chromatography. The mixture containing contaminants was used directly in the next reaction. Removal of the *p*-methoxybenzyl (PMB) group under acidic conditions allowed for purification of the newly formed trisaccharide, affording **14** with a yield of 88% over two steps. Acetylation of the liberated hydroxyl group afforded compound **15** with a yield of 95%. Next, the coupling reaction of 15 with *N*-Cbz-protected aminopentanol **16** occurred smoothly in the presence of *N*-iodosuccinimide (NIS) and TfOH [44,45] in CH_2_Cl_2_ at 0 °C to give the desired glycoside **17** in 85% yield. Subsequent deprotection of the Troc group by treatment with zinc and AcOH [46] in 1,2-dichloroethane at 40 °C afforded common trisaccharide derivative **18** with a yield of 90%.

For constructing the A and B antigen skeletons, it is necessary to incorporate galactosamine (for A antigen) and galactose (for B antigen) residues into trisaccharide **18** in α-linked form. Typically, α-d-galactosides are obtained by using ethereal solvents such as diethyl ether and 1,4-dioxane as well as the anomeric effect [47].

However, highly α-selectivity in such galactosylation is generally difficult and strongly dependent on various factors, such as the substrate structure, promoter, and temperature. The stereoisomers formed are often difficult to separate, which presents a serious disadvantage for synthetic studies. In 2003, we developed a reliable method for α-selective galactosidation and galactosaminidation using DTBS-protected glycosyl donors [48,49,50,51]. Notable features of the DTBS-directed α-galactosylation are excellent α-selectivity even in the presence of a neighboring participating group on the C2 oxygen or nitrogen, and the relatively greater difference between the *R*_f_ values of the α and β isomers that enables them to be more easily separated. Thus, we decided to utilize DTBS-directed α-galactosylation for the construction of the A and B antigen sequences. 

**Scheme 3:**
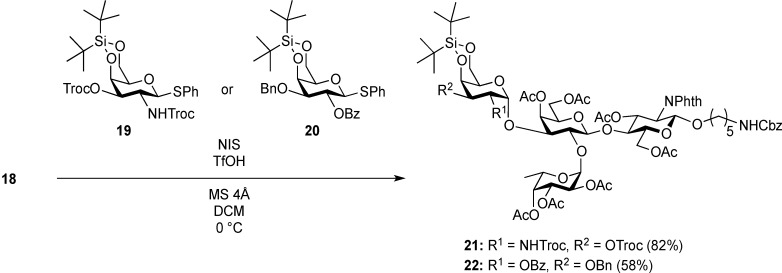
Assembly of A and B antigen sequences.

As shown in Scheme 3, trisaccharide acceptor **18** was glycosylated with galactosaminyl donor **19** [48] and galactosyl donor **20** [52] in the presence of NIS and TfOH in CH_2_Cl_2_ at 0 °C, giving the corresponding tetrasaccharides **21** and **22** in α-linked form in yields of 82% and 58%, respectively. In these reactions, the recovery of unreacted acceptor **18** was 9% and 22%, when **19** and **20** were used, respectively, despite the use of 2 equiv of donor. However, other possible stereoisomers were not detected and both α-products were easy to isolate by column chromatography. To our surprise, the coupling yield of **22** was moderate. When we attempted to use the armed 2,3-di-*O*-benzyl-type galactose donor instead of **20**, the yield was not improved (41%) and many unidentified by-products were generated. The unexpectedly low reactivity of **18** as a glycosyl acceptor might arise from steric hindrance around 3-OH on the Gal residue.

**Scheme 4:**
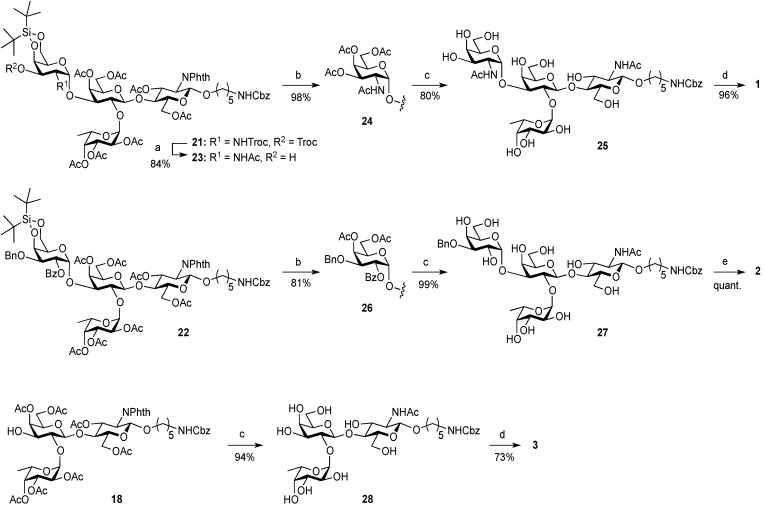
Global deprotection sequence.

*Reagents and conditions*: (**a**) (i) Zn, AcOH, (CH_2_Cl)_2_, 40 °C, (ii) Ac_2_O, MeOH, r.t.; (**b**) (i) TBAHF, THF, r.t. (ii) Ac_2_O, Py, r.t.; (**c**) (i) NH_2_NH_2_·H_2_O, EtOH, reflux, (ii) Ac_2_O, MeOH, r.t.; (**d**) H_2_, Pd/C, MeOH–H_2_O (1:1), r.t.; (**e**) H_2_, Pd/C, 1,4-dioxane–2% aq. formic acid (1:1), r.t.

On the route to the target compounds, there is a global deprotection sequence (Scheme 4). Selective removal of the Troc groups of **21** by treatment with zinc and AcOH, followed by selective acetylation of the liberated amine of the galactosamine residue at C2 afforded **23** in 84% yield. Then, removal of the DTBS group with tributylamine hydrofluoride (TBAHF) in THF [53] followed by acetylation of the hydroxyl groups provided **24** in 98% yield over two steps. After removal of all acetyl groups on **24**, the phthalimide group at C2 of the glucosamine residue was converted to an acetamide group by sequential treatment with hydrazine hydrate in refluxing EtOH followed by selective acetylation of the free amine, affording **25** in 80% yield over three steps. Finally, the Cbz group at the terminus of the linker was removed by hydrogenolysis with Pd/C under hydrogen atmosphere, thus furnishing target **1** (A antigen) in 81% yield. Similarly, the deprotection of compounds **22** and **18** were efficiently carried out to furnish target compounds **2** (B antigen) and **3** (O antigen) in good yields.

## 3. Experimental

### 3.1. General Methods

All reactions were carried out under a positive pressure of argon, unless otherwise noted. All chemicals were purchased from commercial suppliers and used without further purification, unless otherwise noted. Molecular sieves were purchased from Nacalai Tesque, Inc. (Kyoto, Japan) and dried at 300 °C for 12 h in a muffle furnace prior to use. Solvents as reaction media such as CH_2_Cl_2_, MeOH, THF, DMF, and pyridine, which were tapped off from The Solvent Supply System, were purchased from Kanto Chemical Co., Inc. (Tokyo, Japan) and used without purification. TLC analysis was performed on Merck TLC (silica gel 60F254 on glass plate, Darmstadt, Germany). Compound detection was either by exposure to UV light (2536 Å) or by soak in a solution of 10% H_2_SO_4_ in ethanol followed by heating. Silica gel (80 mesh and 300 mesh) manufactured by Fuji Silysia Chemical Ltd. (Kasugai, Japan) was used for flash column chromatography. Quantity of silica gel was usually estimated as 100 to 200-fold weight of sample to be charged. Solvent systems in chromatography were specified in *v*/*v*. Evaporation and concentration were carried out *in vacuo*. ^1^H-NMR and ^13^C-NMR spectra were recorded with Bruker Biospin AVANCE III 500/800 spectrometers (Billerica, MA, USA). Chemical shifts in ^1^H-NMR spectra are expressed in ppm (δ) relative to the signal of Me_4_Si, adjusted to δ 0.00 ppm. Data are presented as follow: Chemical shift, multiplicity (s = singlet, d = doublet, t = triplet, dd = double of doublet, td = triple doublet, m = multiplet and/or multiple resonances), integration, coupling constant in Hertz (Hz), position of the corresponding proton. COSY methods were used to confirm the NMR peak assignments. High-resolution mass (ESI-TOF MS) spectra were run in a Bruker Daltonics micrOTOF (Billerica, MA, USA). Optical rotations were measured with a ‘Horiba SEPA-300’ high-sensitive polarimeter (Kyoto, Japan).

### 3.2. Physical Data for All New Compounds


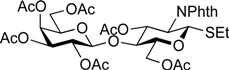


*Ethyl (2,3,4,6-tetra-O-acetyl-β-d-galactopyranosyl)-(1→4)-3,6-di-O-acetyl-2-deoxy-2-phthalimide-1-thio-β-d-glucopyranoside* (**6**)*.* To a mixture of **5** (4.16 g, 5.44 mmol) in (CH_2_Cl)_2_ (27.2 mL) were added EtSH (606 µL, 8.16 mmol) and BF_3_·OEt_2_ (1.03 mL, 8.16 mmol) at 0 °C. After stirring for 2 h at rt as the reaction was monitored by TLC (3:2 EtOAc–hexane), the reaction was quenched by the addition of crushed ice. The solution was diluted with CHCl_3_ and subsequently washed with ice-cooled H_2_O, satd aq Na_2_CO_3_, and brine. The organic layer was then dried over Na_2_SO_4_, and concentrated. The resulting residue was purified by silica gel column chromatography (1:1 EtOAc–hexane) to give **6** (3.99 g, 96%). Spectroscopic data of **6** were identical to those reported in the literature [54]. 


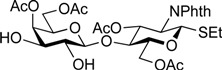


*Ethyl (4,6-di-O-acetyl-β-d-galactopyranosyl)-(1→4)-3,6-di-O-acetyl-2-deoxy-2-phthalimide-1-thio-β-d-glucopyranoside* (**10**)*.* To a solution of **6** (1.08 g, 1.41 mmol) in MeOH/CH_2_Cl_2_ (2:1, 14.1 mL) was added NaOMe (28% solution in MeOH, 31.9 µL, 141 µmol) at 0 °C. After stirring for 2 h at rt as the reaction was monitored by TLC (3:2 EtOAc–hexane), the reaction was neutralized with AcOH. After concentration, the resulting residue was diluted with CHCl_3_ and subsequently washed with H_2_O and brine. The organic layer was dried over Na_2_SO_4_, of which solid was filtered through cotton and the filtrate was then evaporated (giving **7**). The residue was subjected to next reaction without further purification. The crude product **7** was dissolved in MeOH (28.2 mL). To the solution were added 2,3-butanedione (492 µL, 5.64 mmol), trimethyl orthoformate (1.95 mL, 17.8 mmol), and (±)-10-camphorsulfonic acid (66 mg, 282 µmol) at rt. After stirring for 20 h at reflux as the reaction was monitored by TLC (10:1 CHCl_3_–MeOH), the reaction was quenched by the addition of triethylamine (218 µmol) and concentrated. The resulting residue was diluted with CHCl_3_ and subsequently washed with H_2_O and brine. The organic layer was dried over Na_2_SO_4_, filtered, concentrated. The resulting residue was roughly purified by silica gel column chromatography (20:1 CHCl_3_–MeOH) to give 2,3-*O*-BDA-protected product **8** along with small amounts of contaminants. The crude mixture (494 mg) was dissolved in pyridine (7.8 mL). To the solution were added Ac_2_O (890 µL, 9.42 mmol) and a catalytic amount of DMAP at 0 °C. After stirring for 1 h at rt as the reaction was monitored by TLC (3:2 EtOAc–hexane), the mixture was co-evaporated with toluene. The resulting residue was diluted with EtOAc and subsequently washed with 2 M HCl, H_2_O, satd aq NaHCO_3_, and brine, dried over Na_2_SO_4_, and concentrated. The resulting residue was purified by silica gel column chromatography (2:3 EtOAc–hexane) to give **9** (634 mg), to which suspension in H_2_O (1.6 mL) was added trifluoroacetic acid (14.4 mL) at 0 °C. After stirring for 2 h at rt as the reaction was monitored by TLC (1:1 CHCl_3_–acetone), the mixture was diluted with toluene and concentrated. The resulting residue was purified by silica gel column chromatography (7:3 CHCl_3_–acetone) to give **10** (548 mg, 57% over four steps). [α]_D_ +18.3° (c 1.0, CHCl_3_); ^1^H-NMR (500 MHz, CDCl_3_) δ 7.88–7.74 (m, 4H, Phth), 5.84 (dd, 1H, *J*_3,4_ = 8.2 Hz, *J*_2,3_ = 11.2 Hz, H-3*^GlcN^*), 5.50 (d, 1H, *J*_1,2_ = 10.6 Hz, H-1*^GlcN^*), 5.28 (d, 1H, *J*_3,4_ = 3.3 Hz, H-4*^Gal^*), 4.63 (dd, 1H, *J*_5,6a_ = 1.6 Hz, *J*_gem_ = 11.9 Hz, H-6a*^GlcN^*), 4.40–4.29 (m, 3H, H-2*^GlcN^*, H-6b*^GlcN^*, H-1*^Gal^*), 4.10–3.98 (m, 2H, H-6a*^Gal^*, H-6b*^Gal^*), 3.91–3.80 (m, 3H, H-4*^GlcN^*, H-5*^GlcN^*, H-5*^Gal^*), 3.75–3.73 (m, 1H, H-3*^Gal^*), 3.62 (d, 1H, *J*_2,OH_ = 3.2 Hz, OH), 3.57–3.53 (m, 1H, H-2*^Gal^*), 3.21 (d, 1H, *J*_3,OH_ = 3.1 Hz, OH), 2.73–2.61 (m, 2H, SC*H_2_*CH_3_), 2.18–1.90 (4 s, 12H, Ac), 1.22 (t, 3H, SCH_2_C*H_3_*); ^13^C-NMR (125 MHz, CDCl_3_) δ 171.2, 170.9, 170.5, 170.0, 167.7, 167.4, 134.4, 134.2, 131.6, 131.2, 123.7, 123.6, 103.1, 81.1, 72.0, 71.9, 71.7, 71.0, 68.7, 63.1, 61.5, 53.9, 29.7, 29.2, 24.6, 21.0, 20.7, 20.7, 20.6, 14.9. HRMS (ESI) *m/z*: found [M+Na]^+^ 706.1776, C_30_H_37_NO_15_S calcd for [M+Na]^+^ 706.1773. 


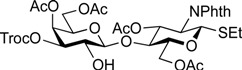


*Ethyl* [4,6-di-O-acetyl-3-O-(2,2,2-trichloroethoxycarbonyl)-β-d-galactopyranosyl]*-(1→4)-3,6-di-O-acetyl-2-deoxy-2-phthalimide-1-thio-β-d-glucopyranoside* (**11**)*.* A solution of **10** (103 mg, 151 µmol) and dibutyltin dichloride (4.6 mg, 15.1 µmol) in acetone (3.0 mL) was stirred for 10 min at rt. To the solution were added PEMP (55 µL, 302 µmol) and TrocCl (27 µL, 196 µmol) at 10 °C. After stirring for 20 min at the same temperature as the reaction was monitored by TLC (1:2 EtOAc–toluene, 1:1 CHCl_3_–acetone), the reaction was quenched by the addition of satd aq NH_4_Cl and concentrated. The resulting residue was diluted with EtOAc and subsequently washed with H_2_O and brine. The organic layer was dried over Na_2_SO_4_, filtered, concentrated. The resulting residue was purified by silica gel column chromatography (2:7 EtOAc–toluene) to give **11** (108 mg, 84%). [α]_D_ +30.0° (c 1.0, CHCl_3_); ^1^H-NMR (500 MHz, CDCl_3_) δ 7.88–7.74 (m, 4H, Phth), 5.70 (dd, 1H, *J*_3,4_ = 8.2 Hz, *J*_2,3_ = 10.6 Hz, H-3*^GlcN^*), 5.49 (d, 1H, *J*_1,2_ = 10.6 Hz, H-1*^GlcN^*), 5.46 (d, 1H, *J*_3,4_ = 2.9 Hz, H-4*^Gal^*), 4.79 (m, 2H, H-3*^Gal^*, OC*H_2_*CCl_3_), 4.65 (near dd, 1H, *J*_gem_ = 11.4 Hz, H-6a*^GlcN^*), 4.44 (d, 1H, *J*_1,2_ = 7.7 Hz, H-1*^Gal^*), 4.38 (dd, 1H, *J*_5,6b_ = 4.2 Hz, H-6b*^GlcN^*), 4.31 (t, 1H, H-2*^GlcN^*), 4.11–4.03 (m, 2H, H-6a*^Gal^*, H-6b*^Gal^*), 3.91–3.77 (m, 4H, H-4*^GlcN^*, H-5*^GlcN^*, H-2*^Gal^*, H-5*^Gal^*), 3.47 (d, 1H, *J*_2,OH_ = 5.2 Hz, OH), 2.72–2.62 (m, 2H, SC*H_2_*CH_3_), 2.14–1.91 (4 s, 12H, Ac), 1.22 (t, 3H, SCH_2_C*H_3_*); ^13^C-NMR (125 MHz, CDCl_3_) δ 171.2, 170.4, 170.2, 170.2, 167.8, 167.5, 153.2, 134.3, 134.3, 131.8, 131.3, 123.8, 103.3, 94.1, 81.2, 77.7, 77.3, 77.2, 72.2, 70.6, 69.2, 66.2, 63.1, 61.1, 54.0, 29.8, 24.6, 21.0, 20.7, 20.7, 20.6, 15.1. HRMS (ESI) *m/z*: found [M+Na]^+^ 880.0823, C_33_H_38_Cl_3_NO_17_S calcd for [M+Na]^+^ 880.0818.


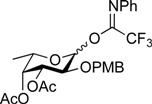


*3,4-Di-O-acetyl-2-O-p-methoxybenzyl-l-fucopyranosyl N-phenyl 2,2,2-trifluoroacetimidate* (**12**). To a solution of phenyl 3,4-di-*O*-acetyl-2-*O*-*p*-methoxybenzyl-1-thio-β-l-fucopyranoside [42] (1.21 g, 2.63 mmol) in acetone/H_2_O (13.1 mL, 96:4) was added NBS (701 mg, 3.94 mmol) at −15 °C. After stirring for 1 h at the same temperature as the reaction was monitored by TLC (1:1 EtOAc–hexane), the reaction was quenched by the addition of satd aq Na_2_S_2_O_3_ and then diluted with EtOAc, washed with H_2_O and brine. The organic layer was dried over Na_2_SO_4_, filtered, concentrated. The resulting residue was purified by silica gel column chromatography (2:3 EtOAc–hexane) to give the corresponding hemiacetal product (969 mg, quant.), which was then dissolved in acetone (52.6 mL). To the solution were added 2,2,2-trifluoro-*N*-phenylacetimidoyl chloride (853 µL, 5.26 mmol) and K_2_CO_3_ (1.82 g, 13.2 mmol) at rt. After stirring for 2.5 h at rt as the reaction was monitored by TLC (1:2 EtOAc–hexane), the reaction mixture was filtered through Celite. The filtrate and washings were concentrated. The resulting residue was purified by silica gel column chromatography (1:4 EtOAc–hexane) to give 12 (1.33 g, 94%, α/β = 1/1). [α]_D_ −81.6° (c 1.0, CHCl_3_); ^13^C-NMR (125 MHz, CDCl_3_) δ 170.3, 170.2, 169.9, 169.8, 159.4, 159.3, 143.5, 143.2, 129.8, 129.7, 129.5, 129.3, 129.1, 128.9, 128.8, 128.6, 128.6, 128.4, 128.4, 124.3, 124.2, 119.3, 119.2, 117.2, 114.9, 114.0, 113.7, 113.7, 97.0, 93.6, 77.6, 77.2, 74.9, 74.7, 72.9, 72.6, 72.2, 70.8, 70.2, 70.0, 69.7, 67.3, 55.2, 55.1, 20.7, 20.6, 20.5, 20.5, 15.9, 15.8. ^1^H-NMR (500 MHz, CDCl_3_) **α-isomer**: δ 7.45–6.71 (m, 9H, Ar), 6.46 (br s, 1H, H-1), 5.35–5.31 (m, 2H, H-3, H-4), 4.75–4.59 (m, 2H, OC*H_2_*Ar), 4.27 (br s, 1H, H-5), 3.95 (br d, 1H, H-2), 3.87–3.77 (m, 3H, OMe), 2.16–1.99 (m, 6H, Ac), 1.18–1.14 (m, 3H, H-6). **β-isomer**: δ 7.45–6.71 (m, 9H, Ar), 5.68 (br s, 1H, H-1), 5.20 (br s, 1H, H-4), 4.98 (br s, 1H, H-3) 4.75–4.59 (m, 2H, OC*H_2_*Ar), 3.87–3.77 (m, 5H, H-2, H-5, OMe), 2.16–1.99 (m, 6H, Ac), 1.18–1.14 (m, 3H, H-6). Possible other stereoisomers were not assigned. HRMS (ESI) *m/z*: found [M+Na]^+^ 562.1657, C_26_H_28_F_3_NO_8_ calcd for [M+Na]^+^ 562.1659.


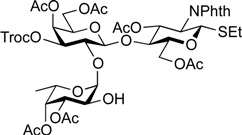


*Ethyl (3,4-di-O-acetyl-α-l-fucopyranosyl)-(1→2)*-[4,6-di-O-acetyl-3-O-(2,2,2-trichloroethoxycarbonyl)-β-d-galactopyranosyl]*-(1→4)-3,6-di-O-acetyl-2-deoxy-2-phthalimide-1-thio-β-d-glucopyranoside* (**14**)*.* To a mixture of **11** (1.06 g, 1.24 mmol) and **12** (1.33 g, 2.47 mmol) in CPME/CH_2_Cl_2_ (1:1, 74.2 mL) was added 4 Å molecular sieves AW-300 (7.42 g) at rt. After stirring for 30 min, the mixture was cooled to −80 °C. TMSOTf (22 µL, 124 µmol) was then added to the mixture at −80 °C. After stirring for 5.5 h at the same temperature as the reaction was monitored by TLC (1:2 EtOAc–toluene, 1:2 EtOAc–hexane) and MALDI-TOF MS, the reaction was quenched by the addition of satd aq NaHCO_3_. The reaction mixture was diluted with CHCl_3_ and filtered through Celite. The filtrate was then washed with satd aq NaHCO_3_ and H_2_O. The organic layer was subsequently dried over Na_2_SO_4_, and concentrated. The resulting residue was purified by silica gel column chromatography (2:7 EtOAc–toluene) to give **13** with unidentified impurity (1.66 g). The crude mixture was then dissolved in CH_2_Cl_2_ (44.6 mL). To the solution was added trifluoroacetic acid (5.0 mL) at 0 °C. After stirring for 40 min at rt as the reaction was monitored by TLC (1:1 EtOAc–hexane), the mixture was co-evaporated with toluene. The residue was diluted with CHCl_3_ and subsequently washed with satd aq NaHCO_3_ and H_2_O. The organic layer was dried over Na_2_SO_4_, filtered, concentrated. The resulting residue was purified by silica gel column chromatography (1:2 EtOAc–toluene) to give **14** (1.18 g, 88% over two steps). [α]_D_ −38.1° (c 1.0, CHCl_3_); ^1^H-NMR (500 MHz, CDCl_3_) δ 7.88–7.73 (m, 4H, Phth), 5.80 (t, 1H, *J*_2,3_ = *J*_3,4_ = 10.6 Hz, H-3*^GlcN^*), 5.47–5.45 (m, 2H, H-1*^GlcN^*, H-4*^Gal^*), 5.29 (d, 1H, *J*_1,2_ = 2.5 Hz, H-1*^Fuc^*), 5.21 (d, 1H, *J*_3,4_ = 3.9 Hz, H-4*^Fuc^*), 4.99 (dd, 1H, *J*_2,3_ = 10.7 Hz, H-3*^Fuc^*), 4.91 (dd, 1H, *J*_3,4_ = 3.6 Hz, *J*_2,3_ = 10.1 Hz, H-3*^Gal^*), 4.75 (s, 2H, OC*H_2_*CCl_3_), 4.51 (dd, 1H, *J*_5,6a_ = 4.2 Hz, *J*_gem_ = 12.0 Hz, H-6a*^GlcN^*), 4.33–4.31 (m, 4H, H-2*^GlcN^*, H-6b*^GlcN^*, H-1*^Gal^*, H-5*^Fuc^*), 4.17–4.09 (m, 2H, H-6a*^Gal^*, H-6b*^Gal^*), 3.95–3.83 (m, 5H, H-4*^GlcN^*, H-5*^GlcN^*, H-2*^Gal^*, H-5*^Gal^*, H-2*^Fuc^*), 2.74–2.62 (m, 2H, SC*H_2_*CH_3_), 2.16–1.91 (6 s, 18H, Ac), 1.27–1.22 (m, 6H, H-6*^Fuc^*, SCH_2_C*H_3_*); ^13^C-NMR (125 MHz, CDCl_3_) δ 170.6, 170.5, 170.3, 169.9, 169.8, 167.5, 167.2, 152.8, 134.3, 134.2, 131.6, 131.2, 123.6, 100.1, 99.6, 93.8, 81.4, 77.8, 77.2, 74.9, 73.2, 71.2, 70.7, 70.6, 67.0, 66.6, 65.7, 62.5, 60.9, 53.9, 29.6, 24.8, 20.8, 20.7, 20.6, 20.6, 20.5, 20.4, 15.6, 15.0. HRMS (ESI) *m/z*: found [M+Na]^+^ 1110.1609, C_43_H_52_Cl_3_NO_23_S calcd for [M+Na]^+^ 1110.1611.


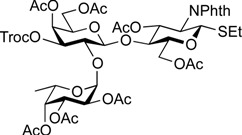


*Ethyl (2,3,4-tri-O-acetyl-α-l-fucopyranosyl)-(1→2)*-[4,6-di-O-acetyl-3-O-(2,2,2-trichloroethoxycarbonyl)-β-d-galactopyranosyl]-*(1→4)-3,6-di-O-acetyl-2-deoxy-2-phthalimide-1-thio-β-d-glucopyranoside* (**15**)*.* To a solution of **14** (1.06 g, 975 µmol) in pyridine (4.9 mL) was added acetic anhydride (4.9 mL) at 0 °C. After stirring for 2 h at rt as the reaction was monitored by TLC (1:1 EtOAc–hexane), the reaction was quenched by addition of MeOH at 0 °C and then evaporated. The residue was diluted with CHCl_3_, washed with 2 M HCl, H_2_O, satd aq NaHCO_3_, and brine, dried over Na_2_SO_4_, concentrated. The residue obtained was purified by silica gel column chromatography (2:3 EtOAc–hexane) to give **15** (1.05 g, 95%). [α]_D_ −39.6° (c 1.0, CHCl_3_); ^1^H-NMR (500 MHz, CDCl_3_) δ 7.86–7.73 (m, 4H, Phth), 5.79 (t, 1H, *J*_2,3_ = *J*_3,4_ = 10.1 Hz, H-3*^GlcN^*), 5.47 (d, 1H, *J*_1,2_ = 10.6 Hz, H-1*^GlcN^*), 5.43 (d, 1H, *J*_3,4_ = 4.3 Hz, H-4*^Gal^*), 5.39 (d, 1H, *J*_1,2_ = 3.8 Hz, H-1*^Fuc^*), 5.34 (d, 1H, *J*_3,4_ = 3.9 Hz, H-4*^Fuc^*), 5.16 (dd, 1H, *J*_2,3_ = 10.9 Hz, H-3*^Fuc^*), 5.07 (dd, 1H, H-2*^Fuc^*), 4.88 (dd, 1H, *J*_2,3_ = 9.8 Hz, H-3*^Gal^*), 4.84 (d, 1H, *J*_gem_ = 11.7 Hz, OC*H_2_*CCl_3_), 4.63 (d, 1H, OC*H_2_*CCl_3_), 4.51 (dd, 1H, *J*_5,6a_ = 1.8 Hz, *J*_gem_ = 10.8 Hz, H-6a*^GlcN^*), 4.47–4.43 (m, 2H, H-1*^Gal^*, H-5*^Fuc^*), 4.39–4.30 (m, 2H, H-2*^GlcN^*, H-6b*^GlcN^*), 4.16 (dd, 1H, *J*_5,6a_ = 6.6 Hz, *J*_gem_ = 11.2 Hz, H-6a*^Gal^*), 4.09 (dd, 1H, H-6b*^Gal^*), 3.94 (t, 1H, H-4*^GlcN^*), 3.89–3.83 (m, 3H, H-5*^GlcN^*, H-2*^Gal^*, H-5*^Gal^*), 2.74–2.62 (m, 2H, SC*H_2_*CH_3_), 2.17–1.91 (7 s, 21H, Ac), 1.26–1.22 (m, 6H, H-6*^Fuc^*, SCH_2_C*H_3_*); ^13^C-NMR (125 MHz, CDCl_3_) δ 170.6, 170.5, 170.3, 170.1, 169.9, 169.7, 169.7, 167.5, 167.2, 152.7, 134.3, 134.1, 131.6, 131.2, 123.6, 100.0, 96.2, 93.8, 81.4, 77.8, 77.2, 76.9, 74.7, 72.5, 71.1, 70.7, 70.6, 67.9, 67.7, 66.4, 65.3, 62.7, 60.9, 53.8, 29.6, 24.8, 20.8, 20.6, 20.6, 20.5, 20.3, 15.5, 15.1. HRMS (ESI) *m/z*: found [M+Na]^+^ 1152.1716, C_45_H_54_Cl_3_NO_24_S calcd for [M+Na]^+^ 1152.1714. 


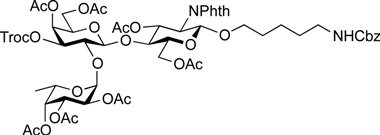


5-Benzyloxycarbonylamino-1-pentyl (2,3,4-tri-O-acetyl-α-l-fucopyranosyl)-(1→2)-[4,6-di-O-acetyl-3-O-(2,2,2-trichloroethoxycarbonyl)-β-d-galactopyranosyl]-(1→4)-3,6-di-O-acetyl-2-deoxy-2-phthalimide-β-d-glucopyranoside (**17**). A mixture of **15** (372 mg, 329 µmol) and **16** (234 mg, 988 µmol), and NIS (148 mg, 658 µmol) was exposed to high vacuum for 1 h. The mixture was dissolved in CH_2_Cl_2_ (13.2 mL), to which 4 Å molecular sieves (1.32 g) was added at rt. After stirring for 30 min at rt and then for 10 min at 0 °C, TfOH (7.1 µL, 65.8 µmol) was added to the mixture. After stirring for 1 h at 0 °C as the reaction was monitored by TLC (1:1 EtOAc–hexane, 2:1 EtOAc–hexane), additional portions of NIS (148 mg, 658 µmol) and TfOH (7.1 µL, 65.8 µmol) were added to the mixture. After 8 h and 16 h, further portions of TfOH (7.1 µL of each) were added to the mixture and the stirring was continued. After stirring for total 30 h, the reaction was quenched by the addition of satd aq NaHCO_3_. The precipitate was filtered through Celite. The filtrate was diluted with CHCl_3_, washed with satd aq Na_2_S_2_O_3_ and brine. The organic layer was subsequently dried over Na_2_SO_4_, concentrated and the residue was then purified by silica gel column chromatography (1:1 EtOAc–hexane) and gel filtration column chromatography (LH-20, 1:1 CHCl_3_–MeOH) to give **17** (375 mg, 87%). [α]_D_ −41.1° (c 1.0, CHCl_3_); ^1^H-NMR (500 MHz, CDCl_3_) δ 7.85–7.69 (m, 4H, Phth), 7.47–7.30 (m, 5H, Ph), 5.74 (dd, 1H, *J*_3,4_ = 9.0 Hz, *J*_2,3_ = 10.8 Hz, H-3*^GlcN^*), 5.42 (d, 1H, *J*_3,4_ = 3.1 Hz, H-4*^Gal^*), 5.40 (d, 1H, *J*_1,2_ = 3.8 Hz, H-1*^Fuc^*), 5.33 (d, 1H, *J*_3,4_ = 3.8 Hz, H-4*^Fuc^*), 5.31 (d, 1H, *J*_1,2_ = 8.5 Hz, H-1*^GlcN^*), 5.17 (dd, 1H, *J*_2,3_ = 10.9 Hz, H-3*^Fuc^*), 5.07–5.04 (m, 3H, H-2*^Fuc^*, OCH_2_), 4.88 (dd, 1H, *J*_2,3_ = 9.8 Hz, H-3*^Gal^*), 4.84 (d, 1H, *J*_gem_ = 11.6 Hz, OCH_2_), 4.64–4.62 (m, 2H, OCH_2_(CH_2_)_3_CH_2_N*H*, OCH_2_), 4.55 (dd, 1H, *J*_5,6a_ = 1.8 Hz, *J*_gem_ = 12.1 Hz, H-6a*^GlcN^*), 4.47–4.43 (m, 2H, H-1*^Gal^*, H-5*^Fuc^*), 4.37 (dd, 1H, *J*_5,6b_ = 5.2 Hz, H-6b*^GlcN^*), 4.24 (dd, 1H, H-2*^GlcN^*), 4.16 (dd, 1H, *J*_5,6a_ = 6.7 Hz, *J*_gem_ = 11.2 Hz, H-6a*^Gal^*), 4.09 (dd, 1H, H-6b*^Gal^*), 3.94 (t, 1H, H-4*^GlcN^*), 3.88–3.79 (m, 4H, H-5*^GlcN^*, H-2*^Gal^*, H-5*^Gal^*, OC*H_2_*(CH_2_)_3_CH_2_NH), 3.46–3.44 (m, 1H, OC*H_2_*(CH_2_)_3_CH_2_NH), 2.95–2.91 (m, 2H, OCH_2_(CH_2_)_3_C*H_2_*NH), 2.17–1.91 (7 s, 21H, Ac), 1.51–1.11 (m, 9H, H-6*^Fuc^*, OCH_2_(C*H_2_*)*_3_*CH_2_NH); ^13^C-NMR (125 MHz, CDCl_3_) δ 170.7, 170.6, 170.3, 170.2, 170.0, 169.8, 156.2, 152.8, 136.7, 134.3, 128.5, 128.1, 128.1, 123.6, 100.1, 98.1, 96.2, 93.8, 77.6, 74.8, 72.9, 72.5, 71.1, 70.6, 70.6, 70.0, 69.8, 67.9, 67.8, 66.5, 66.4, 65.3, 62.3, 61.0, 54.7, 40.8, 29.3, 28.8, 23.0, 20.9, 20.7, 20.6, 20.6, 20.4, 15.5. HRMS (ESI) *m/z*: found [M+Na]^+^ 1327.2890, C_56_H_67_Cl_3_N_2_O_27_ calcd for [M+Na]^+^ 1327.2889.


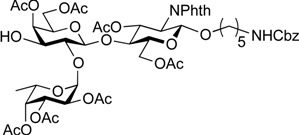


5-Benzyloxycarbonylamino-1-pentyl (2,3,4-tri-O-acetyl-α-l-fucopyranosyl)-(1→2)-(4,6-di-O-acetyl-β-d-galactopyranosyl)-(1→4)-3,6-di-O-acetyl-2-deoxy-2-phthalimide-β-d-glucopyranoside (**18**). To a solution of **17** (289 mg, 215 µmol) in AcOH/(CH_2_Cl)_2_ (3:1, 14.3 mL) was added Zn powder (2.89 g) at rt. The reaction mixture was stirred for 1 h at 40 °C as the reaction was monitored by TLC (3:1 EtOAc–hexane). The precipitate was filtered through Celite and the filtrate was co-evaporated with toluene. The residue obtained was purified by silica gel column chromatography (3:1 EtOAc–hexane) to give **18** (233 mg, 97%). [α]_D_ −56.6° (c 1.0, CHCl_3_); ^1^H-NMR (500 MHz, CDCl_3_) δ 7.85–7.69 (m, 4H, Phth), 7.38–7.30 (m, 5H, Ph), 5.74 (dd, 1H, *J*_3,4_ = 9.0 Hz, *J*_2,3_ = 10.8 Hz, H-3*^GlcN^*), 5.39 (d, 1H, *J*_3,4_ = 3.6 Hz, H-4*^Gal^*), 5.33 (d, 1H, *J*_3,4_ = 3.8 Hz, H-4*^Fuc^*), 5.31 (d, 1H, *J*_1,2_ = 8.5 Hz, H-1*^GlcN^*), 5.26–5.23 (m, 2H, H-3*^Fuc^*, OC*H_2_*Ph), 5.16 (dd, 1H, *J*_1,2_ = 3.6 Hz, *J*_2,3_ = 9.9 Hz, H-2*^Fuc^*), 5.07 (m, 2H, H-1*^Fuc^*, OC*H_2_*Ph), 4.65 (s, 1H, OCH_2_(CH_2_)_3_CH_2_N*H*), 4.51 (dd, 1H, *J*_5,6a_ = 1.8 Hz, *J*_gem_ = 12.0 Hz, H-6a*^GlcN^*), 4.42–4.37 (m, 2H, H-6b*^GlcN^*, H-5*^Fuc^*), 4.31 (d, 1H, *J*_1,2_ = 7.7 Hz, H-1*^Gal^*), 4.23 (dd, 1H, H-2*^GlcN^*), 4.11 (m, 2H, H-6a*^Gal^*, H-6b*^Gal^*), 3.92 (t, 1H, *J*_4,5_ = 9.0 Hz, H-4*^GlcN^*), 3.86–3.79 (m, 4H, H-5*^GlcN^*, H-3*^Gal^*, H-5*^Gal^*, OC*H_2_*(CH_2_)_3_CH_2_NH), 3.54 (dd, 1H, *J*_2,3_ = 9.5 Hz, H-2*^Gal^*), 3.47–3.43 (m, 1H, OC*H_2_*(CH_2_)_3_CH_2_NH), 2.94–2.90 (m, 2H, OCH_2_(CH_2_)_3_C*H_2_*NH), 2.18–1.91 (7 s, 21H, Ac), 1.49–1.11 (m, 9H, H-6*^Fuc^*, OCH_2_(C*H_2_*)*_3_*CH_2_NH); ^13^C-NMR (125 MHz, CDCl_3_) δ 171.0, 170.7, 170.7, 170.4, 170.1, 170.1, 169.9, 156.2, 136.6, 134.3, 131.4, 128.5, 128.0, 123.5, 100.1, 98.1, 97.8, 74.9, 73.0, 72.4, 71.1, 71.0, 69.9, 69.7, 69.6, 68.2, 67.7, 66.5, 65.2, 62.4, 61.5, 54.8, 40.8, 29.6, 29.3, 28.8, 23.0, 20.8, 20.7, 20.6, 20.6, 20.5, 15.7. HRMS (ESI) *m/z*: found [M+Na]^+^ 1153.3847, C_53_H_66_N_2_O_25_ calcd for [M+Na]^+^ 1153.3851.


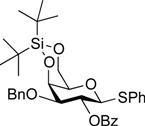


*Phenyl 2-O-benzoyl-3-O-benzyl-4,6-O-di-tert-butylsilylene-1-thio-β-d-galactopyranoside* (**20**). To a solution of Phenyl 3-*O*-benzyl-1-thio-*β*-d-galactopyranoside (262 mg, 724 µmol) in pyridine (7.2 mL) was added di-*tert*-butylsilyl bis(trifluoromethanesulfonate) (260 µL, 796 µmol) at 0 °C. After stirring for 3.5 h at 0 °C as the reaction was monitored by TLC (1:1 EtOAc–hexane), benzoic anhydride (328 mg, 1.45 mmol) was added to the mixture at 0 °C. After stirring for 22 h at rt as the reaction was monitored by TLC (1:3 EtOAc–hexane), the reaction was quenched by the addition of MeOH at 0 °C. The mixture was co-evaporated with toluene. The residue obtained was diluted with EtOAc, washed with 2 M HCl, H_2_O, satd aq NaHCO_3_, and brine, dried over Na_2_SO_4_, concentrated. The resulting residue was purified by silica gel column chromatography (1:7 EtOAc–hexane) to give **20** (324 mg, 74%). [α]_D_ +66.9° (c 0.6, CHCl_3_); ^1^H-NMR (500 MHz, CDCl_3_) δ 8.06–7.16 (m, 15H, Ph), 5.70 (t, 1H, *J*_1,2_ = *J*_2,3_ = 9.8 Hz, H-2), 4.79 (d, 1H, H-1), 4.72 (d, 1H, *J*_gem_ = 12.8 Hz, OC*H_2_*Ph), 4.60–4.57 (m, 2H, H-4, OC*H_2_*Ph), 4.30–4.23 (m, 2H, H-6a, H-6b), 3.57 (dd, 1H, H-3), 3.40 (s, 1H, H-5), 1.16–1.08 (2 s, 18H, 2 *t*-Bu); ^13^C-NMR (125 MHz, CDCl_3_) δ 165.4, 137.9, 134.4, 133.0, 132.1, 130.1, 129.9, 128.8, 128.3, 127.6, 127.5, 87.6, 79.3, 75.1, 70.0, 69.8, 69.4, 67.3, 27.6, 23.4, 20.7. HRMS (ESI) *m/z*: found [M+Na]^+^ 629.2363, C_34_H_42_O_6_SSi calcd for [M+Na]^+^ 629.2364.


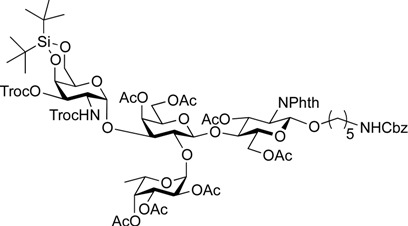


*5-Benzyloxycarbonylamino-1-pentyl* [2-deoxy-4,6-O-di-tert-butylsilylene-2-(2,2,2-trichloroethoxycarbamoyl)-3-O-(2,2,2-trichloroethoxycarbonyl)-α-d-galactopyranosyl]-(1→3)-[2,3,4-tri-O-acetyl-α-l-fucopyranosyl-(1→2)]*-(4,6-di-O-acetyl-β-d-galactopyranosyl)-(1→4)-3,6-di-O-acetyl-2-deoxy-2-phthalimide-β-d-glucopyranoside* (**21**). A mixture of **18** (103 mg, 91.1 µmol) and **19** (138 mg, 182 µmol), and NIS (46 mg, 364 µmol) was exposed to high vacuum for 1 h. The mixture was dissolved in CH_2_Cl_2_ (2.7 mL), to which 4 Å molecular sieves (273 mg) was added at rt. After stirring for 30 min at rt and then for 10 min at 0 °C, TfOH (1.9 µL, 18.2 µmol) was added to the mixture. After stirring for 3 h at 0 °C as the reaction was monitored by TLC (3:1 EtOAc–hexane, 1:1 EtOAc–hexane, 1:3 EtOAc–hexane), additional portions of NIS (23 mg) and TfOH (1.0 µL) were added to the mixture and the stirring was continued. After stirring for total 5 h, the reaction was quenched by the addition of satd aq NaHCO_3_. The precipitate was filtered through Celite. The filtrate was diluted with CHCl_3_, washed with satd aq Na_2_S_2_O_3_ and brine. The organic layer was subsequently dried over Na_2_SO_4_, concentrated and the residue was then purified by silica gel column chromatography (1:1 EtOAc–hexane) to give **21** (132 mg, 82%), and 9.5 mg (9%) of **18** was recovered. [α]_D_ +23.8° (c 1.7, CHCl_3_); ^1^H-NMR (500 MHz, CD_3_CN) δ 7.77–7.70 (m, 4H, Phth), 7.30–7.22 (m, 5H, Ph), 5.74 (d, 1H, *J*_NH,2_ = 9.7 Hz, NH*^GalN^*), 5.64 (dd, 1H, *J*_3,4_ = 9.0 Hz, *J*_2,3_ = 11.9 Hz, H-3*^GlcN^*), 5.35 (d, 1H, *J*_3,4_ = 2.8 Hz, H-4*^Gal^*), 5.30 (m, 2H, H-1*^Fuc^*, OCH_2_(CH_2_)_3_CH_2_N*H*), 5.24 (d, 1H, *J*_3,4_ = 2.3 Hz, H-4*^Fuc^*), 5.20 (d, 1H, *J*_1,2_ = 10.8 Hz, H-1*^GlcN^*), 5.08 (d, 1H, *J*_1,2_ = 4.0 Hz, H-1*^GalN^*), 5.07 (dd, 1H, *J*_1,2_ = 3.5 Hz, *J*_2,3_ = 11.0 Hz, H-2*^Fuc^*), 4.97 (dd, 1H, H-3*^Fuc^*), 4.93 (s, 2H, OCH_2_), 4.86–4.79 (m, 2H, OCH_2_), 4.78–4.68 (m, 3H, H-3*^GalN^*, H-4*^GalN^*, OCH_2_), 4.59 (d, 1H, *J*_gem_ = 12.3 Hz, OCH_2_), 4.41–4.28 (m, 6H, H-6a*^GlcN^*, H-1*^Gal^*, H-5*^Fuc^*, H-2*^GalN^*, H-6a*^GalN^*, H-6b*^GalN^*), 4.09–3.96 (m, 5H, H-2*^GlcN^*, H-4*^GlcN^*, H-6b*^GlcN^*, H-3*^Gal^*, H-6a*^Gal^*), 3.92 (dd, 1H, *J*_5,6b_ = 6.1 Hz, *J*_gem_ = 11.3 Hz, H-6b*^Gal^*), 3.80–3.75 (m, 3H, H-5*^GlcN^*, H-5*^Gal^*, H-5*^GalN^*), 3.65–3.61 (m, 2H, H-2*^Gal^*, OC*H_2_*(CH_2_)_3_CH_2_NH), 3.39–3.34 (m, 1H, OC*H_2_*(CH_2_)_3_CH_2_NH), 2.71–2.65 (m, 2H, OCH_2_(CH_2_)_3_C*H_2_*NH), 2.18–1.80 (7 s, 21H, Ac), 1.31–0.96 (m, 27H, H-6*^Fuc^*, 2 *t*-Bu, OCH_2_(C*H_2_*)*_3_*CH_2_NH); ^13^C-NMR (125 MHz, CD_3_CN) δ 171.6, 171.5, 171.3, 171.2, 171.1, 155.5, 154.2, 135.7, 132.3, 129.4, 128.8, 128.7, 118.6, 118.3, 101.2, 98.9, 97.5, 96.6, 95.5, 94.4, 94.4, 79.1, 77.5, 76.6, 75.2, 74.4, 74.1, 73.5, 71.9, 71.5, 71.3, 70.8, 70.4, 69.0, 68.9, 68.6, 67.3, 66.6, 66.5, 65.7, 63.2, 62.2, 55.5, 49.4, 41.3, 30.0, 29.5, 27.9, 27.8, 23.7, 23.7, 21.5, 21.3, 21.2, 21.1, 21.0, 21.0, 20.8, 16.1. HRMS (ESI) *m/z*: found [M+Na]^+^ 1802.3642, C_73_H_95_Cl_6_N_3_O_33_Si calcd for [M+Na]^+^ 1802.3640.


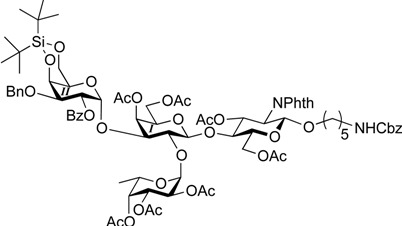


5-Benzyloxycarbonylamino-1-pentyl (2-O-benzoyl-3-O-benzyl-4,6-O-di-tert-butylsilylene-α-d-galactopyranosyl)-(1→3)-[2,3,4-tri-O-acetyl-α-l-fucopyranosyl-(1→2)]-(4,6-di-O-acetyl-β-d-galactopyranosyl)-(1→4)-3,6-di-O-acetyl-2-deoxy-2-phthalimide-β-d-glucopyranoside (**22**). A mixture of **18** (49.7 mg, 44.0 µmol) and **20** (53.3 mg, 87.9 µmol), and NIS (22.0 mg, 176 µmol) was exposed to high vacuum for 1 h. The mixture was dissolved in CH_2_Cl_2_ (1.3 mL), to which 4 Å molecular sieves (132 mg) was added at rt. After stirring for 30 min at rt and then for 10 min at 0 °C, TfOH (1.0 µL, 8.79 µmol) was added to the mixture. After stirring for 1.5 h at 0 °C as the reaction was monitored by TLC (3:1 EtOAc–hexane, 1:1 EtOAc–hexane, 1:3 EtOAc–hexane), additional portion of TfOH (1.0 µL) was added to the mixture and the stirring was continued. After stirring for total 2 h, the reaction was quenched by the addition of satd aq NaHCO_3_. The precipitate was filtered through Celite. The filtrate was diluted with CHCl_3_, washed with satd aq Na_2_S_2_O_3_ and brine. The organic layer was subsequently dried over Na_2_SO_4_, concentrated and the residue was then purified by silica gel column chromatography (7:8 EtOAc–hexane) to give **22** (41.2 mg, 58%), and 10.8 mg (22%) of **18** was recovered. [α]_D_ +43.5° (c 1.3, CHCl_3_); ^1^H-NMR (500 MHz, CDCl_3_) δ 7.96–7.21 (m, 19H, Ar), 5.70 (dd, 1H, *J*_3,4_ = 8.7 Hz, *J*_2,3_ = 10.9 Hz, H-3*^GlcN^*), 5.64 (dd, 1H, *J*_1,2_ = 3.6 Hz, *J*_2,3_ = 10.4 Hz, H-2*^GalII^*), 5.52 (d, 1H, *J*_3,4_ = 2.3 Hz, H-4*^Fuc^*), 5.41–5.40 (m, 2H, H-4*^GalI^*, H-1*^GalII^*), 5.34–5.33 (m, 2H, H-1*^GlcN^*, H-1*^Fuc^*), 5.13–5.07 (m, 4H, H-2*^Fuc^*, H-3*^Fuc^*, OC*H_2_*Ph), 4.83 (d, 1H, *J*_3,4_ = 2.1 Hz, H-4*^GalII^*), 4.75 (d, 1H, *J*_gem_ = 12.0 Hz, OC*H_2_*Ph), 4.63 (br s, 1H, OCH_2_(CH_2_)_3_CH_2_N*H*), 4.54 (d, 1H, OC*H_2_*Ph), 4.47 (dd, 1H, *J*_5,6a_ = 3.9 Hz, *J*_gem_ = 12.2 Hz, H-6a*^GlcN^*), 4.41 (d, 1H, H-6b*^GlcN^*), 4.37–4.19 (m, 5H, H-2*^GlcN^*, H-1*^GalI^*, H-6a*^GalI^*, H-6b*^GalI^*, H-5*^Fuc^*), 4.13 (dd, 1H, *J*_5,6a_ = 6.7 Hz, *J*_gem_ = 11.3 Hz, H-6a*^GalII^*), 3.98–3.90 (m, 3H, H-4*^GlcN^*, H-3*^GalII^*, H-6b*^GalII^*), 3.86–3.78 (m, 3H, H-5*^GlcN^*, H-3*^GalI^*, OC*H_2_*(CH_2_)_3_CH_2_NH), 3.69–3.63 (m, 3H, H-2*^GalI^*, H-5*^GalI^*, H-5*^GalII^*), 3.47–3.45 (m, 1H, OC*H_2_*(CH_2_)_3_CH_2_NH), 2.93–2.89 (m, 2H, OCH_2_(CH_2_)_3_C*H_2_*NH), 2.25–1.81 (6 s, 18H, Ac), 1.47–1.11 (m, 30H, H-6*^Fuc^*, 2 *t*-Bu, Ac, OCH_2_(C*H_2_*)*_3_*CH_2_NH); ^13^C-NMR (125 MHz, CDCl_3_) δ 170.6, 170.6, 170.4, 170.1, 170.0, 169.8, 169.2, 169.3, 165.7, 138.4, 136.6, 134.3, 133.0, 131.4, 130.2, 129.8, 128.5, 128.2, 128.1, 128.1, 127.5, 127.4, 123.5, 100.9, 98.1, 95.9, 92.7, 77.6, 74.3, 72.6, 71.1, 70.9, 70.3, 69.9, 69.7, 69.6, 68.6, 68.4, 68.1, 67.8, 66.8, 66.5, 65.4, 64.4, 62.3, 61.2, 54.6, 40.8, 29.7, 29.3, 28.8, 27.7, 27.3, 23.4, 23.0, 20.9, 20.8, 20.7, 20.6, 20.6, 19.5, 15.9. HRMS (ESI) *m/z*: found [M+Na]^+^ 1649.6129, C_81_H_102_N_2_O_31_Si calcd for [M+Na]^+^ 1649.6128.


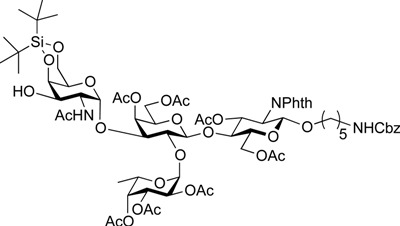


5-Benzyloxycarbonylamino-1-pentyl (2-acetamido-2-deoxy-4,6-O-di-tert-butylsilylene-α-d-galactopyranosyl)-(1→3)-[2,3,4-tri-O-acetyl-α-l-fucopyranosyl-(1→2)]-(4,6-di-O-acetyl-β-d-galactopyranosyl)-(1→4)-3,6-di-O-acetyl-2-deoxy-2-phthalimide-β-d-glucopyranoside (**23**). To a solution of **21** (45 mg, 25.2 µmol) in CH_2_Cl_2_ (1.7 mL) were added AcOH (288 µL, 5.04 mmol) and Zn powder (225 mg, 3.44 mmol) at rt. After stirring for 20 min at rt as the reaction was monitored by TLC (20:1 CHCl_3_–MeOH), another portion of Zn powder (225 mg) was added to the mixture and the stirring was continued. After 30 min, AcOH (288 µL) and CH_2_Cl_2_ (1.7 mL) were added to the mixture. After stirring for total 4 h, the precipitate was filtered through Celite and the filtrate was washed with satd aq NaHCO_3_. The organic layer was subsequently dried over Na_2_SO_4_, concentrated and the residue obtained was then dissolved in CH_2_Cl_2_ (2.5 mL). To the mixture was added acetic anhydride (48 µL, 252 µmol) at 0 °C. After stirring for 1 h at rt as the reaction was monitored by TLC (2:1 CHCl_3_–acetone), the reaction mixture was concentrated. The resulting residue was purified by silica gel column chromatography (2:1 CHCl_3_–acetone) to give **23** (31 mg, 84%). [α]_D_ +6.3° (c 0.6, CHCl_3_); ^1^H-NMR (500 MHz, CDCl_3_) δ 7.85–7.70 (m, 4H, Phth), 7.38–7.26 (m, 5H, Ph), 5.76–5.69 (m, 2H, H-3*^GlcN^*, NH*^GalN^*), 5.45–5.43 (m, 2H, H-4*^Gal^*, H-4*^Fuc^*), 5.36–5.31 (m, 2H, H-1*^GlcN^*, H-2*^Fuc^*), 5.15–5.07 (m, 5H, H-1*^Fuc^*, H-3*^Fuc^*, H-1^GalN^, OCH_2_), 4.63 (br s, 1H, OCH_2_(CH_2_)_3_CH_2_N*H*), 4.50–4.43 (m, 4H, H-2*^GlcN^*, H-6a*^GlcN^*, H-6b*^GlcN^*, H-4*^GalN^*), 4.41–4.35 (m, 2H, H-1*^Gal^*, H-5*^Fuc^*), 4.29 (d, 1H, *J*_gem_ = 11.2 Hz, H-6a*^GalN^*), 4.25–4.18 (m, 2H, H-2^GalN^, H-6b*^GalN^*), 4.10–4.04 (m, 2H, H-6a*^Gal^*, H-6b*^Gal^*), 3.95–3.92 (t, 1H, *J*_3,4_ = *J*_4,5_ = 9.9 Hz, H-4*^GlcN^*), 3.89–3.72 (m, 5H, H-5*^GlcN^*, H-2*^Gal^*, H-3*^Gal^*, H-5*^Gal^*, OC*H_2_*(CH_2_)_3_CH_2_NH), 3.56–3.45 (m, 3H, H-3^GalN^, H-5^GalN^, OC*H_2_*(CH_2_)_3_CH_2_NH), 2.94–2.90 (m, 2H, OCH_2_(CH_2_)_3_C*H_2_*NH), 2.60 (d, 1H, *J*_3,OH_ = 11.5 Hz, OH*^GalN^*), 2.18–1.88 (8 s, 24H, Ac), 1.51–1.05 (m, 27H, H-6*^Fuc^*, 2 *t*-Bu, OCH_2_(C*H_2_*)*_3_*CH_2_NH); ^13^C-NMR (125 MHz, CD_3_CN) δ 170.2, 170.2, 169.9, 169.9, 169.8, 169.7, 169.6, 155.9, 137.3, 134.4, 131.0, 128.1, 127.5, 127.4, 123.1, 117.0, 99.5, 97.6, 95.6, 93.4, 73.8, 73.6, 73.1, 72.6, 72.2, 70.8, 70.4, 69.8, 69.1, 67.8, 67.7, 67.6, 67.5, 66.3, 65.3, 64.8, 64.7, 61.7, 61.1, 54.2, 53.9, 48.6, 40.0, 30.9, 29.0, 28.7, 28.4, 28.3, 26.7, 26.4, 22.5, 22.4, 21.8, 20.2, 19.9, 19.9, 19.8, 19.7, 19.7, 19.6, 19.5, 14.7. HRMS (ESI) *m/z*: found [M+Na]^+^ 1496.5661, C_69_H_95_N_3_O_30_Si calcd for [M+Na]^+^ 1496.5662.


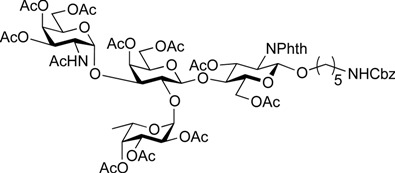


5-Benzyloxycarbonylamino-1-pentyl (2-acetamido-3,4,6-tri-O-acetyl-2-deoxy-α-d-galactopyranosyl)-(1→3)-[2,3,4-tri-O-acetyl-α-l-fucopyranosyl-(1→2)]-(4,6-di-O-acetyl-β-d-galactopyranosyl)-(1→4)-3,6-di-O-acetyl-2-deoxy-2-phthalimide-β-d-glucopyranoside (**24**). To a solution of **23** (31 mg, 21.2 µmol) in THF (1.1 mL) was added TBAHF 1.0 m solution (212 µL) at rt. After stirring for 40 min at rt as the reaction was monitored by TLC (10:1 CHCl_3_–MeOH), the reaction mixture was diluted with EtOAc and washed with 2 M HCl, H_2_O, satd aq NaHCO_3_, and brine. The organic layer was then dried over Na_2_SO_4_ and concentrated. The residue obtained was dissolved in pyridine (1.0 mL). To the mixture was added acetic anhydride (1.0 mL) at 0 °C. After stirring for 21 h at rt as the reaction was monitored by TLC (10:1 CHCl_3_–MeOH), the reaction was quenched by the addition of MeOH at 0 °C. The mixture was co-evaporated with toluene. The residue was diluted with EtOAc and washed with 2 M HCl, H_2_O, satd aq NaHCO_3_, and brine. The organic layer was then dried over Na_2_SO_4_ and concentrated. The resulting residue was purified by silica gel column chromatography (10:10:1 CHCl_3_-toluene-MeOH) and gel filtration column chromatography (LH-20, 1:1 CHCl_3_–MeOH) to give **24** (30 mg, 98% over two steps). [α]_D_ +2.6° (c 1.0, CHCl_3_); ^1^H-NMR (500 MHz, CDCl_3_) δ 7.85–7.70 (m, 4H, Phth), 7.38–7.26 (m, 5H, Ph), 6.32 (br d, 1H, *J*_2,NH_ = 6.9 Hz, NH*^GalN^*), 5.76 (dd, 1H, *J*_2,3_ = 10.8 Hz, *J*_3,4_ = 9.2 Hz, H-3*^GlcN^*), 5.50 (d, 1H, *J*_1,2_ = 3.7 Hz, H-1*^Fuc^*), 5.42 (d, 1H, *J*_3,4_ = 1.5 Hz, H-4*^GalN^*), 5.37–5.34 (m, 3H, H-4*^Gal^*, H-2*^Fuc^*, H-4*^Fuc^*), 5.31 (d, 1H, *J*_1,2_ = 8.5 Hz, H-1*^GlcN^*), 5.22 (d, 1H, *J*_1,2_ = 3.2 Hz, H-1*^GalN^*), 5.13 (dd, 1H, *J*_3,4_ = 3.2 Hz, *J*_2,3_ = 11.0 Hz, H-3*^Fuc^*), 5.07 (s, 2H, OC*H_2_*Ph), 4.97 (dd, 1H, *J*_2,3_ = 11.3 Hz, H-3*^GalN^*), 4.66 (br s, 1H, OCH_2_(CH_2_)_3_CH_2_N*H*), 4.56–4.51 (m, 2H, H-6a*^GlcN^*, H-5*^Fuc^*), 4.48–4.43 (m, 1H, H-2*^GalN^*), 4.40–4.37 (m, 2H, H-6b*^GlcN^*, H-1*^Gal^*), 4.24 (dd, 1H, H-2*^GlcN^*), 4.17–4.14 (m, 2H, H-6a*^Gal^*, H-6a*^GalN^*), 4.11–4.07 (m, 2H, H-6b*^Gal^*, H-6b*^GalN^*), 4.03 (br d, 1H, H-5*^GalN^*), 3.96 (t, 1H, *J*_4,5_ = 9.2 Hz, H-4*^GlcN^*), 3.86–3.74 (m, 5H, H-5*^GlcN^*, H-2*^Gal^*, H-3*^Gal^*, H-5*^Gal^*, OC*H_2_*(CH_2_)_3_CH_2_NH), 3.48–3.44 (m, 1H, OC*H_2_*(CH_2_)_3_CH_2_NH), 2.94–2.90 (m, 2H, OCH_2_(CH_2_)_3_C*H_2_*NH), 2.20–1.92 (11 s, 33H, Ac), 1.49–1.05 (m, 9H, H-6*^Fuc^*, OCH_2_(C*H_2_*)*_3_*CH_2_NH); ^13^C-NMR (125 MHz, CDCl_3_) δ 170.9, 170.6, 170.5, 170.4, 170.3, 170.0, 170.0, 169.9, 136.6, 134.3, 128.5, 128.1, 128.1, 123.6, 100.3, 98.1, 96.6, 77.6, 74.6, 74.5, 72.8, 71.2, 70.6, 69.9, 69.8, 68.6, 67.9, 67.4, 66.9, 66.7, 66.5, 65.3, 62.7, 62.2, 61.0, 54.7, 48.1, 40.8, 29.7, 29.3, 28.8, 23.0, 20.9, 20.7, 20.6, 20.6, 15.6. HRMS (ESI) *m/z*: found [M+Na]^+^ 1482.4956, C_67_H_85_N_3_O_33_Si calcd for [M+Na]^+^ 1482.4958.


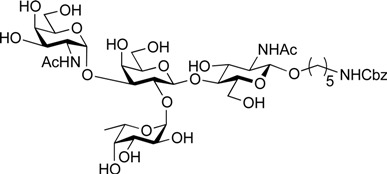


5-Benzyloxycarbonylamino-1-pentyl (2-acetamido-2-deoxy-α-d-galactopyranosyl)-(1→3)-[α-l-fucopyranosyl-(1→2)]-β-d-galactopyranosyl-(1→4)-2-acetamide-2-deoxy-β-d-glucopyranoside (**25**). To a solution of **24** (19.8 mg, 13.6 µmol) in MeOH (1.4 mL) was added NaOMe (1m solution in MeOH, 6.8 µL, 6.78 µmol) at 0 °C. After stirring for 4 h at rt as the reaction was monitored by TLC (20:12:1 CHCl_3_–MeOH–H_2_O), the reaction was neutralized with Muromac (H^+^) resin. The resin was filtered out and the filtrate was concentrated. The residue obtained was then dissolved in EtOH (2.8 mL). To the solution was added NH_2_NH_2_·H_2_O (1.0 µL, 27.2 µmol) at rt. The reaction mixture was stirred at reflux as monitored by TLC (5:4:1 CHCl_3_–MeOH–H_2_O). Additional portions of NH_2_NH_2_·H_2_O (2.0 µL) was added to the mixture every 15 min (total amounts of NH_2_NH_2_·H_2_O added was 32 µL). After 6.5 h, the reaction mixture was concentrated and exposed to high vacuum for 1 h. The resulting residue was then dissolved in MeOH/CH_2_Cl_2_ (3:1, 4.4 mL). To the mixture was added acetic anhydride (26 µL, 272 µmol) at 0 °C. After stirring for 1.5 h at rt as the reaction was monitored by TLC (5:4:1 CHCl_3_-MeOH-H_2_O), the reaction mixture was concentrated. The residue obtained was purified by silica gel column chromatography (Iatrobeads, 9:5:0.5 CHCl_3_–MeOH–H_2_O) to give **25** (10.3 mg, 80% over three steps). [α]_D_ +4.4° (c 0.3, MeOH); ^1^H-NMR (500 MHz, CD_3_OD) δ 7.45–7.43 (m, 5H, Ph), 5.36 (d, 1H, *J*_1,2_ = 3.9 Hz, α-anomer H), 5.15 (d, 1H, *J*_1,2_ = 3.7 Hz, α-anomer H), 5.06 (s, 2H, OC*H_2_*Ph), 4.52 (d, 1H, *J*_1,2_ = 7.7 Hz, β-anomer H), 4.39 (d, 1H, *J*_1,2_ = 8.4 Hz, β-anomer H), 4.34–4.31 (m, 1H, H-5*^Fuc^*), 4.18–3.46 (m, 27H, ring H, OC*H_2_*(CH_2_)_3_CH_2_NH), 3.11–3.08 (m, 2H, OCH_2_(CH_2_)_3_C*H_2_*NH), 2.00–1.96 (2 s, 6H, Ac), 1.57–1.20 (m, 9H, H-6*^Fuc^*, OCH_2_(C*H_2_*)*_3_*CH_2_NH); ^13^C-NMR (125 MHz, CD_3_OD) δ 174.5, 173.5, 158.9, 138.5, 129.4, 128.9, 128.8, 102.8, 102.2, 100.3, 93.6, 78.5, 77.9, 77.2, 76.9, 74.2, 73.6, 73.5, 72.7, 71.9, 70.5, 70.5, 70.1, 69.9, 67.7, 67.3, 64.9, 63.4, 62.6, 61.8, 56.9, 51.3, 41.8, 30.5, 30.2, 24.3, 23.0, 22.7, 16.6. HRMS (ESI) *m/z*: found [M+Na]^+^ 974.3954, C_41_H_65_N_3_O_22_ calcd for [M+Na]^+^ 974.3952.


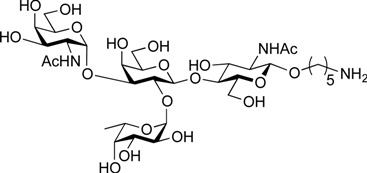


5-Amino-1-pentyl 2-acetamido-2-deoxy-α-d-galactopyranosyl-(1→3)-[α-l-fucopyranosyl-(1→2)]-β-d-galactopyranosyl-(1→4)-2-acetamide-2-deoxy-β-d-glucopyranoside (**1**). To a solution of **25** (3.2 mg, 3.36 µmol) in MeOH/H_2_O (1:1, 3.2 mL) was added Pd/C (5 wt. %, 0.5 mg). After stirring for 3.5 h at rt under a hydrogen atmosphere as the reaction was monitored by TLC (5:4:1:1 CHCl_3_–MeOH–H_2_O–AcOH), additional portion of Pd/C (0.5 mg) was added to the mixture and the stirring was continued. After 12.5 h, further portion of Pd/C (0.5 mg) was added to the mixture. After stirring for total 21 h, the mixture was filtered through membrane filter. The filtrate was concentrated and the residue obtained was purified by gel filtration column chromatography (LH-20, MeOH) to give **1** (2.2 mg, 96%). [α]_D_ +4.4° (c 0.3, MeOH); ^1^H-NMR (500 MHz, D_2_O) δ 5.36 (d, 1H, *J*_1,2_ = 4.1 Hz, α-anomer H), 5.16 (d, 1H, *J*_1,2_ = 3.9 Hz, α-anomer H), 4.58 (d, 1H, *J*_1,2_ = 7.7 Hz, β-anomer H), 4.47 (d, 1H, *J*_1,2_ = 8.4 Hz, β-anomer H), 4.31–4.29 (m, 1H, H-5*^Fuc^*), 4.23–3.56 (m, 27H, ring H, OC*H_2_*(CH_2_)_3_CH_2_NH), 2.98–2.95 (m, 2H, OCH_2_(CH_2_)_3_C*H_2_*NH), 2.02 (2 s, 6H, Ac), 1.67–1.22 (m, 9H, H-6*^Fuc^*, OCH_2_(C*H_2_*)*_3_*CH_2_NH); ^13^C-NMR (200 MHz, CD_3_OD) δ 174.4, 173.6, 103.0, 102.2, 100.3, 93.5, 78.3, 77.8, 77.1, 77.0, 74.1, 73.6, 73.5, 72.7, 71.9, 70.5, 70.3, 70.0, 69.9, 67.7, 64.8, 63.4, 62.6, 61.6, 56.8, 51.2, 40.7, 33.1, 30.8, 30.5, 29.8, 28.3, 24.2, 23.8, 23.0, 22.7, 16.6, 14.5. HRMS (ESI) *m/z*: found [M+Na]^+^ 840.3584, C_33_H_59_N_3_O_20_ calcd for [M+Na]^+^ 840.3584.


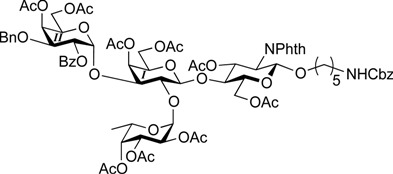


5-Benzyloxycarbonylamino-1-pentyl (4,6-di-O-acetyl-2-O-benzoyl-3-O-benzyl-α-d-galactopyranosyl)-(1→3)-[2,3,4-tri-O-acetyl-α-l-fucopyranosyl-(1→2)]-(4,6-di-O-acetyl-β-d-galactopyranosyl)-(1→4)-3,6-di-O-acetyl-2-deoxy-2-phthalimide-β-d-glucopyranoside (**26**). Compound **22** (30.1 mg, 18.5 µmol) was converted into **26** (23.6 mg, 81%) according to the procedure described for **24**. [α]_D_ +75.3° (c 0.2, CHCl_3_); ^1^H-NMR (500 MHz, CDCl_3_) δ 7.96–7.16 (m, 19H, Ar), 5.75 (d, 1H, *J*_3,4_ = 1.9 Hz, H-4*^GalII^*), 5.72 (dd, 1H, *J*_3,4_ = 8.2 Hz, *J*_2,3_ = 8.8 Hz, H-3*^GlcN^*), 5.56 (d, 1H, *J*_3,4_ = 2.6 Hz, H-4*^Fuc^*), 5.42 (d, 1H, *J*_1,2_ = 3.9 Hz, H-1*^Fuc^*) 5.41 (d, 1H, *J*_3,4_ = 2.3 Hz, H-4*^GalI^*), 5.38–5.35 (m, 2H, H-1*^GalII^*, H-2*^GalII^*), 5.31 (d, 1H, *J*_1,2_ = 8.4 Hz, H-1*^GlcN^*), 5.22–5.17 (m, 2H, H-2*^Fuc^*, H-3*^Fuc^*), 5.07 (s, 2H, OC*H_2_*Ph), 4.70 (d, 1H, *J*_gem_ = 11.8 Hz, OC*H_2_*Ph), 4.64 (br s, 1H, OCH_2_(CH_2_)_3_CH_2_N*H*), 4.49–4.40 (m, 4H, H-6a*^GlcN^*, H-6b*^GlcN^*, H-5*^Fuc^*, OC*H_2_*Ph), 4.30 (d, 1H, *J*_1,2_ = 7.4 Hz, H-1*^GalI^*), 4.23–4.13 (m, 4H, H-2*^GlcN^*, H-5*^GalII^*, H-6a*^GalII^*, H-6b*^GalII^*), 4.08 (dd, 1H, *J*_3,4_ = 3.2 Hz, *J*_2,3_ = 7.2 Hz, H-3*^GalII^*), 4.00 (dd, 1H, *J*_5,6a_ = 6.7 Hz, *J*_gem_ = 11.3 Hz, H-6a*^GalI^*), 3.94–3.90 (m, 2H, H-4*^GlcN^*, H-6b*^GalI^*), 3.86–3.79 (m, 2H, H-5*^GlcN^*, OC*H_2_*(CH_2_)_3_CH_2_NH), 3.76 (dd, 1H, *J*_2,3_ = 7.4 Hz, *J*_3,4_ = 2.9 Hz, H-3*^GalI^*), 3.65 (t, 1H, H-2*^GalI^*), 3.57 (t, 1H, *J*_5,6b_ = 6.7 Hz, H-5*^GalI^*), 3.48–3.43 (m, 1H, OC*H_2_*(CH_2_)_3_CH_2_NH), 2.93–2.89 (m, 2H, OCH_2_(CH_2_)_3_C*H_2_*NH), 2.24–1.83 (9 s, 27H, Ac), 1.47–1.09 (m, 9H, H-6*^Fuc^*, OCH_2_(C*H_2_*)*_3_*CH_2_NH); ^13^C-NMR (125 MHz, CDCl_3_) δ 170.6, 170.6, 170.5, 17.3, 170.2, 170.1, 169.9, 169.8, 169.4, 165.6, 156.2, 137.8, 136.6, 134.3, 133.3, 131.4, 129.9, 129.6, 128.5, 128.4, 128.2, 128.1, 128.1, 127.9, 127.5, 123.5, 100.5, 98.1, 96.1, 77.6, 74.2, 72.7, 71.4, 71.3, 70.9, 70.1, 69.8, 69.6, 68.0, 67.8, 67.7, 67.2, 66.5, 65.2, 62.5, 62.3, 61.2, 54.6, 40.8, 29.7, 29.3, 28.8, 23.0, 20.8, 20.8, 20.7, 20.7, 20.7, 20.6, 19.8, 15.8. HRMS (ESI) *m/z*: found [M+Na]^+^ 1593.4316, C_81_H_102_N_2_O_31_Si calcd for [M+Na]^+^ 1593.4318.


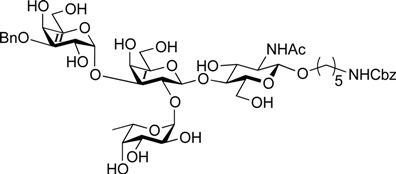


5-Benzyloxycarbonylamino-1-pentyl (3-O-benzyl-α-d-galactopyranosyl)-(1→3)-[α-l-fucopyranosyl-(1→2)]-β-d-galactopyranosyl-(1→4)-2-acetamide-2-deoxy-β-d-glucopyranoside (**27**). Compound **26** (23.3 mg, 14.8 µmol) was converted into **27** (14.7 mg, 99%) according to the procedure described for **25**. [α]_D_ −6.2° (c 0.3, MeOH); ^1^H-NMR (500 MHz, CD_3_OD) δ 7.45–7.26 (m, 10H, Ph), 5.30 (near s, 1H, α-anomer H), 5.15 (d, 1H, *J*_1,2_ = 3.9 Hz, α-anomer H), 5.05 (s, 2H, OC*H_2_*Ph), 4.75 (d, 1H, *J*_gem_ = 11.7 Hz, OC*H_2_*Ph), 4.64 (d, 1H, OC*H_2_*Ph), 4.53 (d, 1H, *J*_1,2_ = 7.5 Hz, β-anomer H), 4.38 (d, 1H, *J*_1,2_ = 8.4 Hz, β-anomer H), 4.29–4.28 (m, 1H, H-5*^Fuc^*), 4.12–3.45 (m, 27H, ring H, OC*H_2_*(CH_2_)_3_CH_2_NH), 3.11–3.08 (m, 2H, OCH_2_(CH_2_)_3_C*H_2_*NH), 1.96 (s, 3H, Ac), 1.56–1.21 (m, 9H, H-6*^Fuc^*, OCH_2_(C*H_2_*)*_3_*CH_2_NH); ^13^C-NMR (125 MHz, CD_3_OD) δ 173.5, 158.9, 139.9, 138.5, 129.4, 129.3, 129.1, 128.9, 128.8, 128.7, 102.8, 102.2, 100.2, 95.9, 79.5, 79.3, 78.6, 77.1, 76.7, 74.1, 73.6, 73.0, 72.6, 71.9, 70.5, 69.9, 69.1, 68.1, 67.6, 67.3, 65.6, 63.3, 62.6, 61.7, 56.7, 41.8, 30.5, 30.2, 24.3, 23.0, 16.6. HRMS (ESI) *m/z*: found [M+Na]^+^ 1023.4156, C_46_H_68_N_2_O_22_ calcd for [M+Na]^+^ 1023.4156.


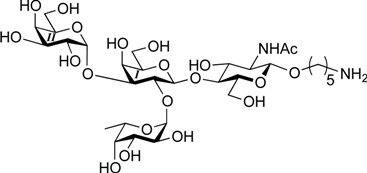


5-Amino-1-pentyl α-d-galactopyranosyl-(1→3)-[α-l-fucopyranosyl-(1→2)]-β-d-galactopyranosyl-(1→4)-2-acetamide-2-deoxy-β-d-glucopyranoside (**2**). Compound **27** (2.1 mg, 2.10 µmol) was converted into **2** (1.6 mg, quant.) according to the procedure described for **1**, except for the use of a mixed solvent (1:1, 1,4-dioxane–2% aq formic acid) as reaction media. [α]_D_ +6.3° (c 0.3, MeOH); ^1^H-NMR (500 MHz, D_2_O) δ 5.31 (d, 1H, *J*_1,2_ = 4.1 Hz, α-anomer H), 5.22 (d, 1H, *J*_1,2_ = 2.5 Hz, α-anomer H), 4.59 (d, 1H, *J*_1,2_ = 7.6 Hz, β-anomer H), 4.46 (d, 1H, *J*_1,2_ = 8.4 Hz, β-anomer H), 4.30–3.42 (m, 28H, ring H, OC*H_2_*(CH_2_)_3_CH_2_NH), 2.98–2.95 (m, 2H, OCH_2_(CH_2_)_3_C*H_2_*NH), 2.01 (s, 3H, Ac), 1.67–1.21 (m, 9H, H-6*^Fuc^*, OCH_2_(C*H_2_*)*_3_*CH_2_NH); ^13^C-NMR (200 MHz, CD_3_OD) δ 173.5, 103.0, 102.2, 100.3, 96.2, 79.9, 78.5, 77.1, 76.7, 74.1, 73.8, 73.6, 73.2, 71.8, 71.4, 71.3, 70.3, 70.0, 69.9, 67.6, 65.8, 63.3, 62.6, 61.7, 56.7, 40.7, 29.8, 28.4, 24.2, 23.0, 16.5. HRMS (ESI) *m/z*: found [M+Na]^+^ 779.3320, C_31_H_56_N_2_O_20_ calcd for [M+Na]^+^ 779.3319.


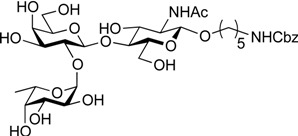


*5-Benzyloxycarbonylamino-1-pentyl* *α-l-fucopyranosyl-(1→2)-β-d-galactopyranosyl-(1→4)-2-acetamide-2-deoxy-β-d-glucopyranoside* (**28**). Compound **18** (19.3 mg, 17.0 µmol) was converted into **28** (12.0 mg, 94%) according to the procedure described for **25**. [α]_D_ −110.0° (c 0.2, MeOH); ^1^H-NMR (500 MHz, CD_3_OD) δ 7.34–7.28 (m, 5H, Ph), 5.22 (d, 1H, *J*_1,2_ = 3.1 Hz, α-anomer H), 5.05 (s, 2H, OC*H_2_*Ph), 4.48 (d, 1H, *J*_1,2_ = 6.1 Hz, β-anomer H), 4.37 (d, 1H, *J*_1,2_ = 8.3 Hz, β-anomer H), 4.18–4.17 (m, 1H, H-5*^Fuc^*), 3.96–3.45 (m, 20H, ring H, OC*H_2_*(CH_2_)_3_CH_2_NH), 3.11–3.08 (m, 2H, OCH_2_(CH_2_)_3_C*H_2_*NH), 1.96 (s, 3H, Ac), 1.57–1.20 (m, 9H, H-6*^Fuc^*, OCH_2_(C*H_2_*)*_3_*CH_2_NH); ^13^C-NMR (125 MHz, CD_3_OD) δ 173.5, 158.9, 138.5, 129.4, 128.9, 128.8, 102.8, 102.5, 101.8, 79.0, 78.2, 77.1, 77.0, 76.9, 75.3, 74.1, 73.6, 71.7, 70.7, 70.5, 68.3, 67.3, 62.6, 61.6, 56.7, 41.8, 30.5, 30.2, 24.3, 23.0, 16.7. HRMS (ESI) *m/z*: found [M+Na]^+^ 771.3156, C_33_H_52_N_2_O_17_ calcd for [M+Na]^+^ 771.3158.


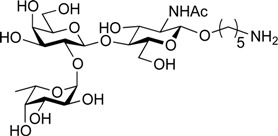


*5-Amino-1-pentyl* *α-l-fucopyranosyl-(1→2)-β-d-galactopyranosyl-(1→4)-2-acetamide-2-deoxy-β-d-glucopyranoside* (**3**). Compound **28** (5.9 mg, 7.88 µmol) was converted into **3** (3.5 mg, 73%) according to the procedure described for **1**. [α]_D_ −76.3° (c 0.2, MeOH); ^1^H-NMR (500 MHz, D_2_O) δ 5.29 (d, 1H, *J*_1,2_ = 3.1 Hz, α-anomer H), 4.52 (d, 1H, *J*_1,2_ = 7.8 Hz, β-anomer H), 4.48 (d, 1H, *J*_1,2_ = 8.2 Hz, β-anomer H), 4.22–4.20 (m, 1H, H-5*^Fuc^*), 3.98–3.42 (m, 18H, ring H, OC*H_2_*(CH_2_)_3_CH_2_NH), 2.98–2.95 (m, 2H, OCH_2_(CH_2_)_3_C*H_2_*NH), 2.02 (s, 3H, Ac), 1.69–1.21 (m, 9H, H-6*^Fuc^*, OCH_2_(C*H_2_*)*_3_*CH_2_NH); ^13^C-NMR (200 MHz, CD_3_OD) δ 173.6, 103.0, 102.5, 101.8, 79.0, 78.0, 77.1, 76.9, 75.2, 74.1, 73.6, 71.7, 70.7, 70.7, 70.2, 68.3, 62.7, 61.5, 56.6, 40.6, 39.5, 29.8, 28.2, 24.1, 23.0, 16.8. HRMS (ESI) *m/z*: found [M+Na]^+^ 637.2791, C_25_H_46_N_2_O_15_ calcd for [M+Na]^+^ 637.2790.

## 4. Conclusions

We have developed a novel approach to synthesizing human histo-blood group type 2 antigens. A lactosamine derivative served as a key building block and was efficiently prepared from lactulose via the Heyns rearrangement, a strategy that allowed us to lower the overall number of reaction steps. The introduction of galactosamine and galactose in α-linked form into the O-antigen trisaccharide was accomplished by a unique DTBS-directed α-glycosylation to afford type 2 A- and B-antigen tetrasaccharides, respectively. The present synthetic protocol can provide rapid access to various biologically relevant glycoconjugates that contain *N*-acetyl-lactosamine and ABO blood group antigens. Studies on biological applications using the synthesized antigens will be reported in due course.
